# Palliative splenic irradiation for symptomatic splenomegaly in non-Hodgkin lymphoma

**DOI:** 10.3332/ecancer.2018.887

**Published:** 2018-12-13

**Authors:** Liliana Castro Oliveira, Carlos Fardilha, Manuel Louro, Carlos Pinheiro, Abílio Sousa, Herlander Marques, Paulo Costa

**Affiliations:** 1 Department of Oncology, Hospital da Senhora da Oliveira, Guimarães, Portugal; 2 Department of Radiation Oncology, Hospital de Braga, Braga, Portugal; 3 Department of Oncology, Hospital de Braga, Braga, Portugal; 4 Clinical Academic Centre, Braga, Portugal; 5 Centre for Health Technology and Services Research, Porto, Portugal

**Keywords:** radiotherapy, splenomegaly, lymphoma, palliative

## Abstract

**Introduction and aims:**

Splenic marginal zone lymphoma, an uncommon subtype of non-Hodgkin lymphoma (NHL), is usually present with symptomatic splenomegaly. Although splenectomy has long been considered the first-line therapy in symptomatic or cytopenic patients, it can lead to significant morbidity and mortality. Splenic irradiation is an option for patients who have a poor response to systemic therapy and/or are not surgical candidates. In this paper, we present a case report of a patient who received splenic radiotherapy for symptomatic splenomegaly.

**Methods:**

An 85-year-old Caucasian man with a 4 year history of low-grade NHL presented with progressive pancytopenia, significant weight loss and symptomatic splenomegaly (abdominal discomfort, sense of fullness and limitation of mobility due to spleen size). The patient refused splenectomy and, in December 2017, was referred to palliative splenic radiotherapy. He was initially treated with five fractions of one Grey (Gy) in order to evaluate clinical and haematology response. After that, 1.5 Gy daily, 5 days a week for 3 weeks. 3D conformal radiotherapy, multiple fields and mixed energy (6 and 15 Mv) were used.

**Results:**

Radiotherapy allowed significant splenic reduction to almost half the size, resolving abdominal discomfort and improving quality of life. There was no decline of haemoglobin, leukocytes and platelet counts; in fact, there was a marginal increase.

**Conclusion:**

Palliative splenic irradiation was well tolerated confirming that it is a safe treatment option for palliation of symptomatic splenomegaly. Thereby, splenic irradiation should be strongly considered in the management of symptomatic splenomegaly, for selected patients who are refractory to or unsuitable for other options or when the patient refuses other treatments.

## Introduction

Non-Hodgkin lymphoma (NHL) is a heterogeneous group of lymphoid malignancies, which develop in the lymph nodes or in other lymph tissue such as the spleen [1]. Splenic marginal zone lymphoma (SMZL), an uncommon subtype of NHL, is usually present with symptomatic splenomegaly [2].

Splenomegaly is a common debilitating complication in lymphoproliferative disorders that may present with abdominal pain, epigastric discomfort, impaired mobility, early satiety and fatigue, and it is often associated with hypersplenism [3, 4]. This condition is caused by an increased demand for splenic function, infiltrative diseases of the spleen and splenic congestion due to portal hypertension and is characterised by splenomegaly, cytopenias, normal/hyperplastic bone marrow and potential response to splenectomy [5].

Some patients may have no criteria for initiating treatment and can be considered for watch and wait. For patients with treatment indication, available therapeutic options are splenectomy, chemotherapy, rituximab alone, rituximab–chemotherapy combination or radiotherapy [6].

Although splenectomy has long been considered the first-line therapy in symptomatic or cytopenic patients, it can lead to significant morbidity and mortality [2, 4].

Splenic irradiation may be an option for patients who have a poor response to systemic therapy and/or are not surgical candidates due to advanced age or poor performance status [7].

In this paper, we present a case report of a patient who received splenic radiotherapy for symptomatic splenomegaly.

## Methods

### Case report

An 85-year-old Caucasian man, with a medical history of deep venous thrombosis, cerebrovascular disease (currently without anticoagulation) and a 4 year history of low-grade NHL (atypical SMZL), presented with progressive pancytopenia, significant weight loss and symptomatic splenomegaly (abdominal discomfort, sense of fullness and limitation of mobility due to spleen size). He was human immunodeficiency virus negative, hepatitis B (HB) antigen negative, anti-HBs antibody positive and hepatitis C virus negative. The patient refused splenectomy and, in December 2017, was referred to palliative splenic radiotherapy.

## Treatment planning

The patient was positioned in a comfortable and reproducible supine position with the arms up using a thorax board, Vac-Lok coach and knee edge.

Simulation was performed using a noncontrast-enhanced computerised tomography (CT), in which images were acquired for every 2-mm CT slice thickness. Four CT scans were used in order to adapt weekly spleen changes and to better protect organs at risk.

At the treatment bed, image verification was done using two setup fields admitting a maximum deviation of 3 mm.

Planning target volume was the spleen with a 1-cm margin, considering setup uncertainties and internal organ movements

3D conformal radiotherapy, multiple fields and mixed energy (6 and 15 Mv) was used. We started with five fractions of one Gy in order to evaluate the patient’s clinical and haematology response. Then, we used 1.5 Gy daily, 5 days a week for 3 weeks. Neither premedication nor concomitant therapy was administered.

The left kidney was the most exposed organ, even though we did not cross international recommendations doses.

## Results

**Figure 1.**
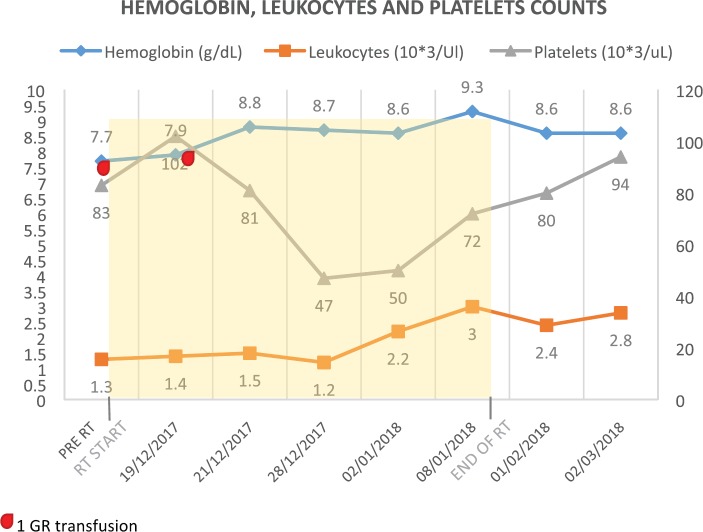
Haemoglobin, leukocytes and platelet counts.

Figure 1 reports the evolution of haemoglobin, leukocytes and platelet counts before, during and after the treatment.

Table 1 shows the evolution of spleen volume before, during and after the treatment.

The treatment was very well tolerated with no adverse events reported.

The patient was observed at 3 and 8 weeks after completing treatment, presenting symptomatic improvement with less abdominal discomfort and restored mobility.

Nine months after the end of spleen irradiation, the patient maintains a satisfactory level of mobility without recurrence of pain and abdominal discomfort. Haemoglobin, leukocytes and platelets counts remain stable. At the time of this publication (December 2018), the patient no more needed re-irradiation.

## Discussion

SMZL is a rare and indolent B-cell NHL that usually presents with symptomatic splenomegaly and lymphocytosis. Sometimes, it is associated with chronic antigenic stimulation by hepatitis C virus. Diagnosis is based on the examination of bone marrow (lymphocyte morphology, immunophenotype, cytogenetic analysis, bone marrow histology) and spleen histology, when available [2].

SMZL is not curable and can be associated with long survival. Asymptomatic patients without splenomegaly, anaemia, thrombocytopenia or leukopenia can only be observed. When patients became symptomatic due to splenomegaly and/or cytopenias, therapy is required. The management of symptomatic patients is determined by several criteria: patient’s performance status [Eastern Cooperative Oncology Group or Karnofsky performance scales]; serology for hepatitis C virus, extent of involvement and patient´s preference. Therapeutic options include systemic therapy (antiviral therapy, if hepatitis C infection; single-agent rituximab; rituximab plus chemotherapy) and local therapies (splenectomy or radiotherapy for patients not candidates or refuse surgery). Although splenectomy remains the treatment of choice for symptomatic splenomegaly, there are patients not eligible for this procedure due to complications such as major bleeding, pulmonary complications, thrombosis and infections [8].

Treatment with chemotherapy, on the other hand, has been associated with considerable morbidity and mortality and less efficacy in elderly patients [9]. Monotherapy with rituximab is well tolerated and induces high response rates in SMZL, but it is especially effective for splenectomised patients and can induce Hepatitis reactivation on HBsAg-negative/anti-HBc-positive patients although with a very low risk in monotherapy [10, 11].

Splenic irradiation, a noninvasive technique, is an effective palliative treatment in patients who are refractory to or unsuitable for surgery or chemotherapy or when the patient refuses other options. It can also increase the response rates to immune or immune-chemotherapy with Rituximab if the patient needs further treatments. In addition, it seems to be safe to repeat irradiation in patients who do not achieve a durable response.

There are several cases described in the literature that show the effectiveness of radiation therapy for the palliative management of symptomatic splenomegaly. Paulino *et al* described 25 patients with lymphoproliferative or myeloproliferative disorders who received splenic irradiation for palliation of splenomegaly and splenic pain with splenomegaly and splenic pain decreased in 60% and 91% of patients, respectively [12]. Weinman *et al*’s review showed that spleen irradiation allowed relief of pain and abdominal discomfort with a response rate of 50%–90% [13]. Similar results were described by Krizetal, reporting pain relief in 74% of the 122 patients analysed [14]. Carolina de la Pinta *et al* presented five patients with symptomatic splenomegaly treated with low doses of radiation (median radiation doses 4.85 Gy; range 2.5–10) and concluded that this approach was effective with significant improvement of splenic pain and abdominal discomfort with a low rate of side effects [15].

Although lower doses can be sufficient for palliation, higher doses are also suggested in the literature (maximum doses of 24 Gy to 32 Gy) [16–19]. In this particular case, we used 27 Gy in order to achieve better hematologic parameters. We were expecting a better hematologic response.

In this case report, radiotherapy allowed significant splenic reduction to almost half the size, resolving abdominal discomfort and improving quality of life. At the end of the treatment, there was no decline of haemoglobin, leukocytes and platelet counts; in fact, there was a marginal increase. Palliative splenic irradiation was well tolerated, confirming that it is a safe treatment option for palliation of symptomatic splenomegaly [3, 20].

## Conclusion

Symptomatic splenomegaly can be safely treated with radiotherapy. Low doses of radiotherapy allow relief of abdominal pain and sense of fullness and recovery of count blood cells improving quality of life. Therefore, splenic irradiation should be strongly considered in the management of symptomatic splenomegaly, for selected patients who are refractory to or unsuitable for other options or when the patient refuses other treatments.

## List of abbreviations

CT: Computerised Tomography
Gy: Grey
HB: Hepatitis B
NHL: Non-Hodgkin Lymphoma
SMZL: Splenic Marginal Zone Lymphoma

## Conflicts of interest

The authors declare no conflicts of interest.

## Ethical approval

All procedures performed respected informed consent and confidentiality.

## Financial disclosure

No financial disclosure.

## Figures and Tables

**Table 1.**
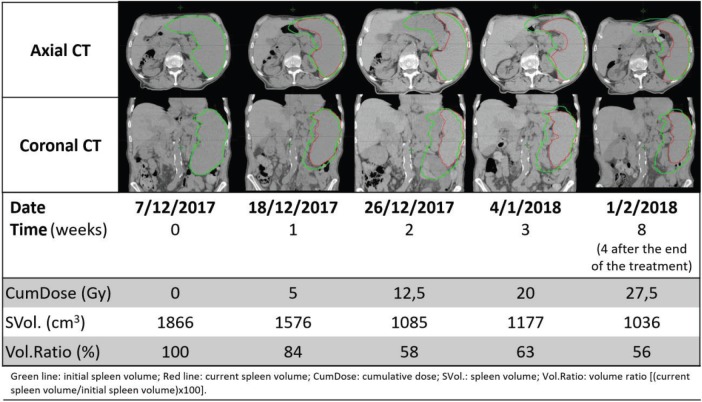
Spleen volume before, during and after the treatment.
